# Representation of female and male pictorial instructions on automated external defibrillators, pad packages, and defibrillation pads

**DOI:** 10.1016/j.resplu.2026.101259

**Published:** 2026-02-11

**Authors:** Nino Fijačko, Robert Greif, Guglielmo Imbriaco, Priyanka Modi, Roos Edgar, Kristin Alm-Kruse, Sergio Cazorla-Calderón

**Affiliations:** a University of Ljubljana, Faculty of Health Sciences, Ljubljana, Slovenia; b Faculty of Medicine, University of Bern, Bern, Switzerland; c 118 Emilia Est Emergency Medical Communication Center, Maggiore Hospital, Bologna, Italy; d Department of Emergency Medicine, SGT Medical College, Hospital and Research Institute, Gurugram, India; e Department of Cardiology, Radboud University Medical Center, Nijmegen, the Netherlands; f Division of Prehospital Services, Oslo University Hospital, Oslo, Norway; g Department of Research and Development, Division of Emergencies and Critical Care, Oslo University Hospital, Oslo, Norway; h University of Vic-Central University of Catalonia. Institute for Research and Innovation in Life Sciences and Health in Central Catalonia, Vic, Spain

**Keywords:** Automated external defibrillator, AED, Pictorial instructions, Cardiac arrest, Resuscitation, Sex, Adult, Neonatal, Pediatric, Female, Male, Girl, Boy

## Abstract

**Objective:**

To evaluate the representation of sex pictograms displayed on automated external defibrillators (AEDs), pad packages, and defibrillation pads, assessing inclusivity and potential implications for real-life AED use.

**Methods:**

Between May and October 2025, investigators from seven countries worldwide captured original photographs of AEDs, pad packages, and defibrillation pads from manufacturers, distributors, and institutional networks. Only adult, commercially available AEDs and defibrillation pads intended for real-life use were included; training or manual AEDs were excluded. Two independent researchers assessed each pictogram for sex representation, with consensus by a third reviewer when needed.

**Results:**

Female pictograms were rare across all reviewed AEDs, pad packages, and defibrillation pad sets. Among 51 AEDs with pictograms, only two (3.9%) included any female pictograms, whereas most displayed only adult male pictograms (62.7%). In pad packages, female pictograms appeared on three packages (7.0%), while 48.8% showed only adult male pictograms. In defibrillation pad sets, female pictograms appeared in two sets (4.5%); most showed only adult male pictograms (56.8%). Neonatal and pediatric pictograms were uncommon overall, with pediatric boy pictograms representing the predominant across AEDs, pad packages, and defibrillation pad sets.

**Conclusions:**

Instructional imagery on AEDs, pad packages, and defibrillation pads overwhelmingly represents male, with minimal or absent female depictions. This imbalance may reinforce gender bias, potentially leading to incorrect pad placement on women and reducing inclusivity in resuscitation. Manufacturers and resuscitation councils could promote accurate and balanced visual representations to ensure equitable and realistic AED use across sexes and body types during cardiac arrest management.

## Graphical abstract

**Figure:**
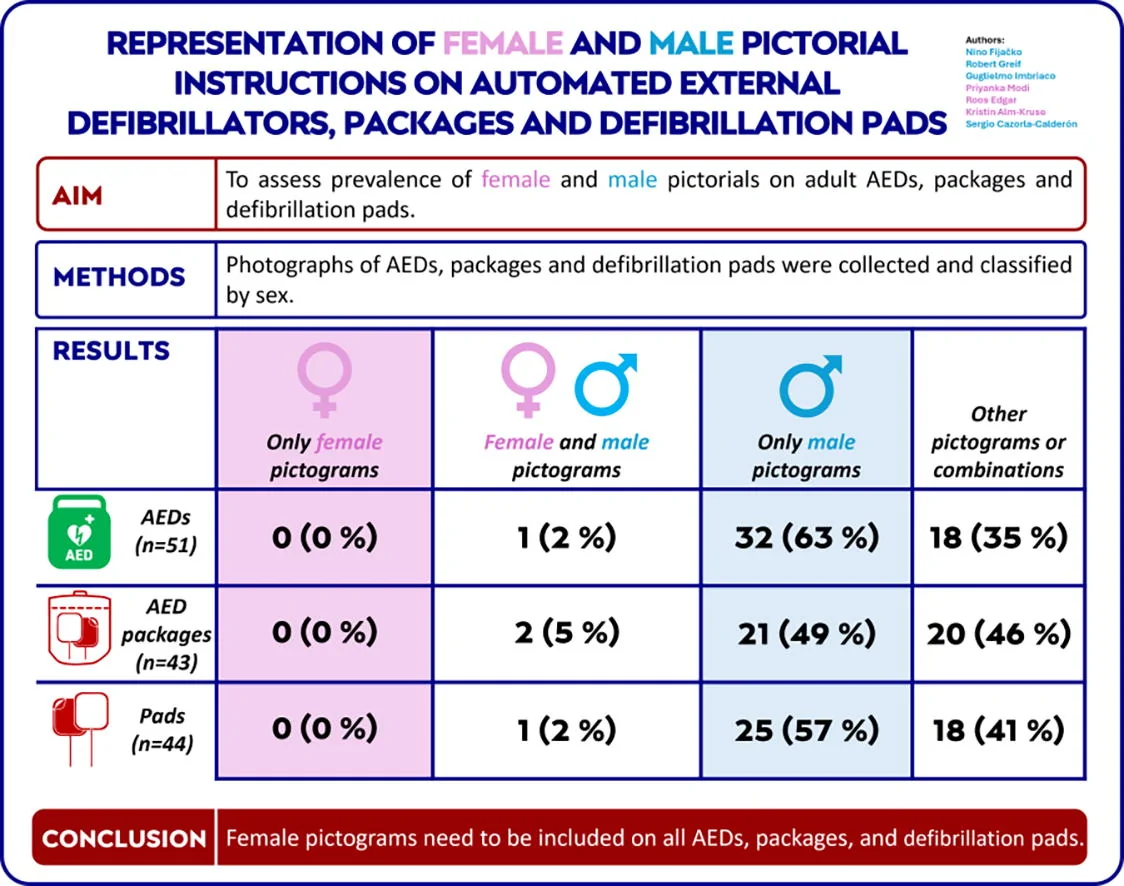

## Introduction

Timely and correct use of automated external defibrillators (AEDs) is critical for improving survival in out-of-hospital cardiac arrest (OHCA) with an initial shockable rhythm. However, AEDs are used correctly and promptly more often in males across both children and adults.^1^, ^2^, ^3^, ^4^, ^5^ In adults specifically, evidence showed that women experiencing OHCA are significantly less likely to have AED pads applied and are less likely to receive a shock when needed.^6^, ^7^, ^8^, ^9^, ^10^, ^11^, ^12^, ^13^, ^14^, ^15^ This unequal treatment may be driven by hesitations or uncertainties, such as societal discomfort with cross-gender contact and the discrepancy between visual aids and real-life scenarios, which can lead to improper AED pad placement or failure to remove clothing such as a bra.^6^, ^7^, ^16^, ^17^

Resuscitation guidelines for adult basic life support (BLS)^18^, ^19^ recommend turning on the AEDs immediately upon arrival and following its prompts. However, studies have shown that AED pads are often placed incorrectly.^20^, ^21^ In addition, current AED pads placement pictograms, a crucial visual aid, have been found to be insufficient to support accurate defibrillation pad placement,^22^ especially in women.^23^

This observational study sought to answer the following research question: “Do instructional pictograms on AEDs, AED pad packages, and defibrillation pad sets include representations of both sexes?”

## Methods

Between May and October 2025, we developed a global database of AEDs using the Google search engine, which included data on the AED brand, company, AEDs, country of origin, and contact information. Investigators from Italy, India, Norway, the Netherlands, Slovenia, Spain, and Switzerland contacted companies, manufacturers, distributors, and their professional networks and local sources to obtain AEDs, pad packages, and defibrillation pad sets for original photographs. For the purpose of this study, “pad packages” was defined as any container, case, box, or pouch supplied with the defibrillation pads. “Defibrillation pad sets” included both sternal (adult)/back (neonatal and pediatric) and apical (adult)/front (neonatal and pediatric) pads. “Pictograms” are visual instructional elements on AEDs, pad packaging, and defibrillation pads, including both static images and short videos that present pad placement on patients.

Our inclusion criteria were AEDs, pad packages, and defibrillation pad sets for adults intended for real-life use and commercially available on the market. Pad packages or defibrillation pad sets designed for manual defibrillators or training purposes, as well as training AEDs, were excluded. To reduce potential sources of bias, only original photographs done by the research group of the AEDs, pad packages, and defibrillation pad sets were allowed; manufacturer stock photographs were not accepted.

We defined a priori which photographs had to be taken from each device. From AEDs the front and back, and the interior if the device had a lid. For AEDs with a display screen, we recorded the video and captured a still photograph showing the sex of the figure during defibrillation pad-placement instruction. Four photographs of each pad package and defibrillation pads were taken: the front and back of the pad package, and the front and back of the defibrillation pads.

Two investigators (NF and SCC) reviewed sex pictograms on the photographs and reached agreement; if consensus was not achieved, a third investigator (KAK) was consulted to resolve discrepancies. Sex pictograms were classified as female or male (adult), girl or boy (pediatric or neonatal) or androgynous based on visible sex attributes such as chest contour, hairstyle, and the presence or absence of breasts. Agreement between investigators who reviewed sex pictograms on the photographs was evaluated using Cohen’s kappa, with interpretation as follows: ≤0 indicating *no agreement*, 0.01–0.20 none to *slight agreement*, 0.21–0.40 fair agreement, 0.41–0.60 *moderate agreement*, 0.61–0.80 *substantial agreement*, and 0.81–1.00 *almost perfect* agreement.^24^

The primary outcome of our study was the representation of pictograms of females and males on AEDs, pad packages, and defibrillation pad sets. The secondary outcome was the representation of neonatal or pediatric (boy or girl) pictograms.

This article was structured following the Strengthening the Reporting of Observational Studies in Epidemiology Statement (Supplementary 1).^25^ Ethics approval was not required for this study, as it did not involve human participants or the use of personal or health-related data. Data was organized and descriptive statistics was calculated using Microsoft Excel 365 (Microsoft Corporation, USA). Results are presented as counts with percentages. The Sankey diagrams were created using SankeyMATIC, a free online tool for making Sankey diagrams and Inkscape v0.35-v.1.4.2 path, free graphics editing software.

## Results

### Representation of sex in AED pictograms

**Fig. 1:**
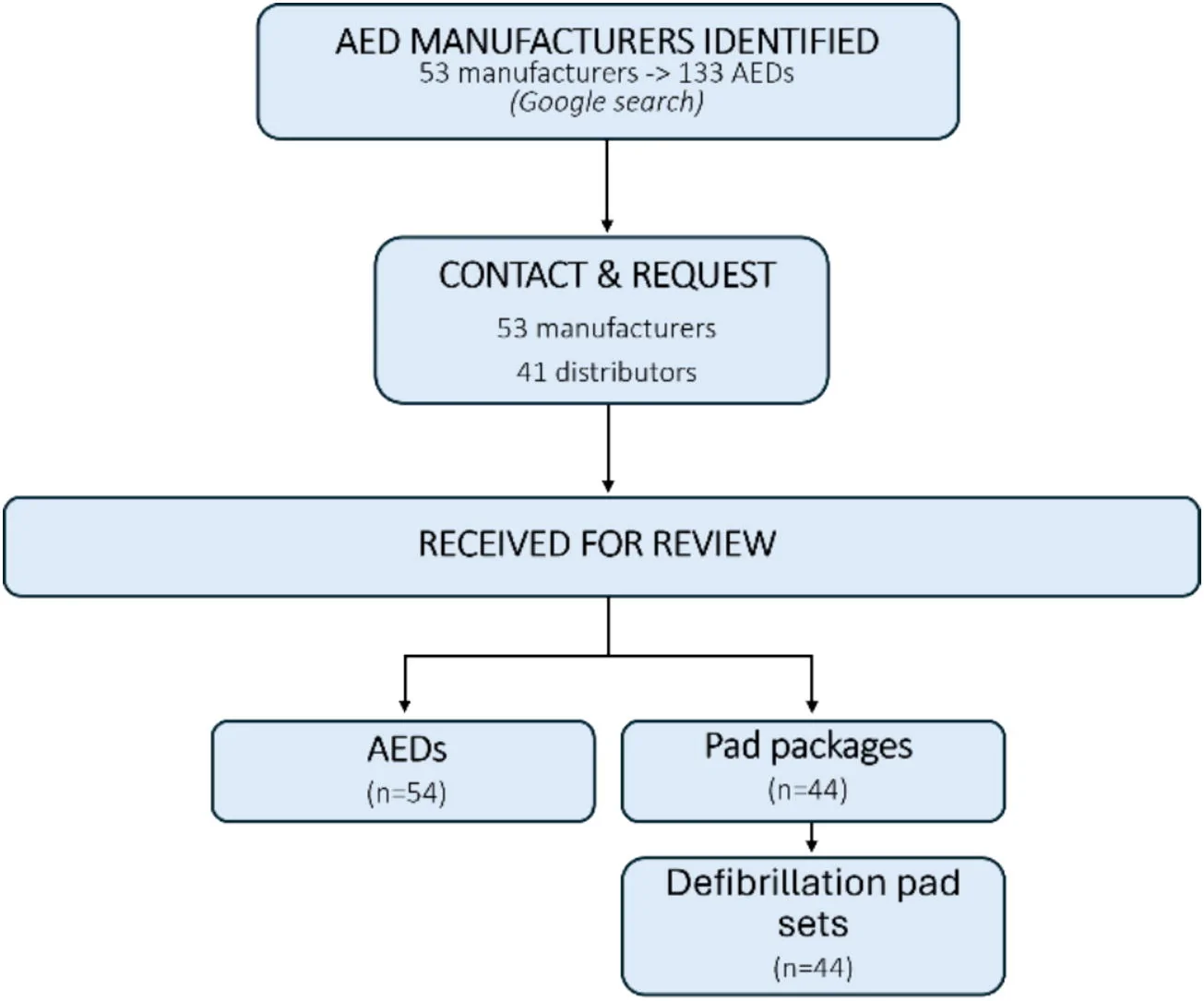
**Flow chart of AEDs, pad packages and defibrillation pad sets sample identification and inclusion process**.

**Fig. 2:**
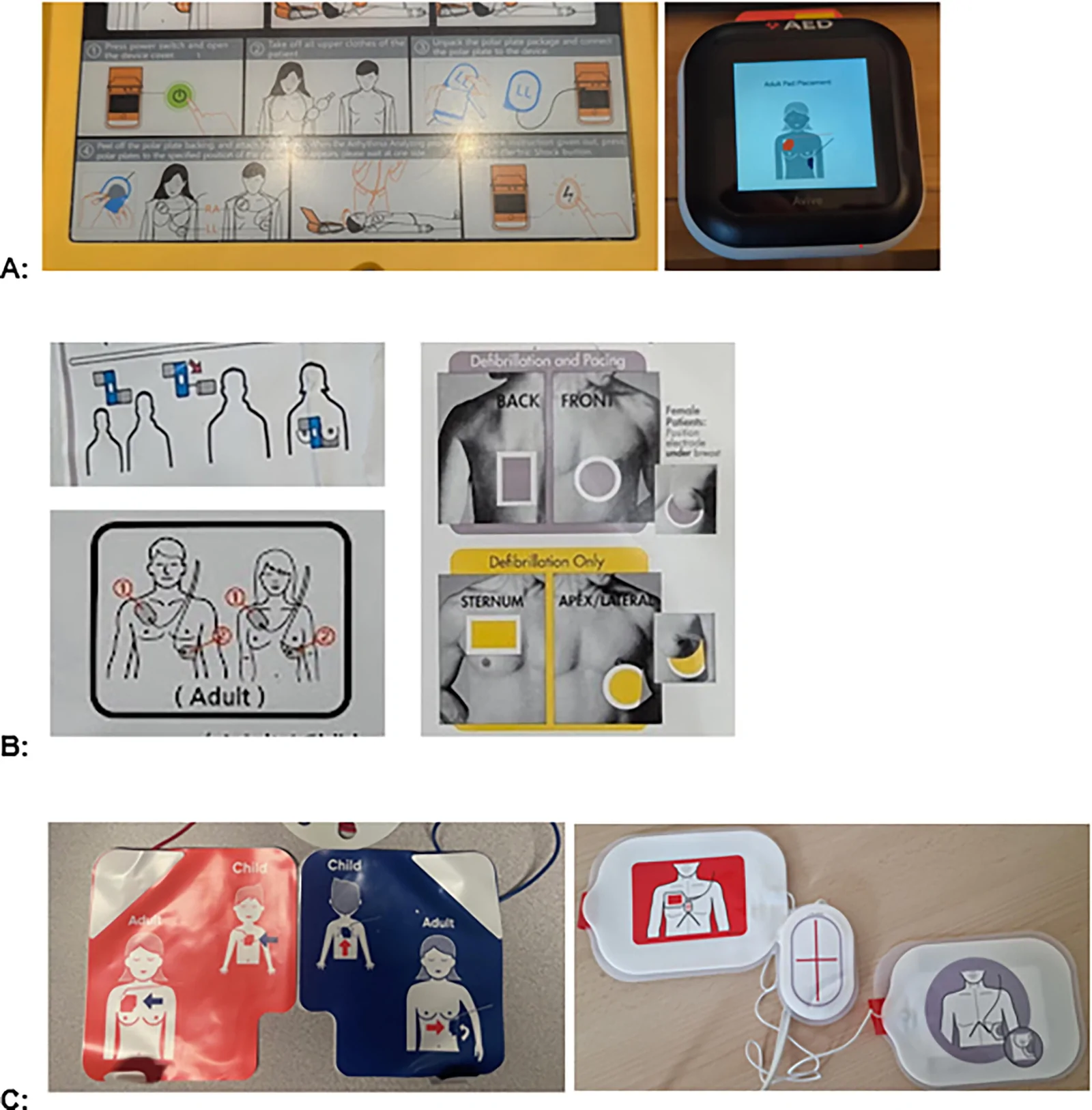
**AEDs (A), pad packages (B), and defibrillation pad sets (C) displaying sex pictograms**.

**Fig. 3:**
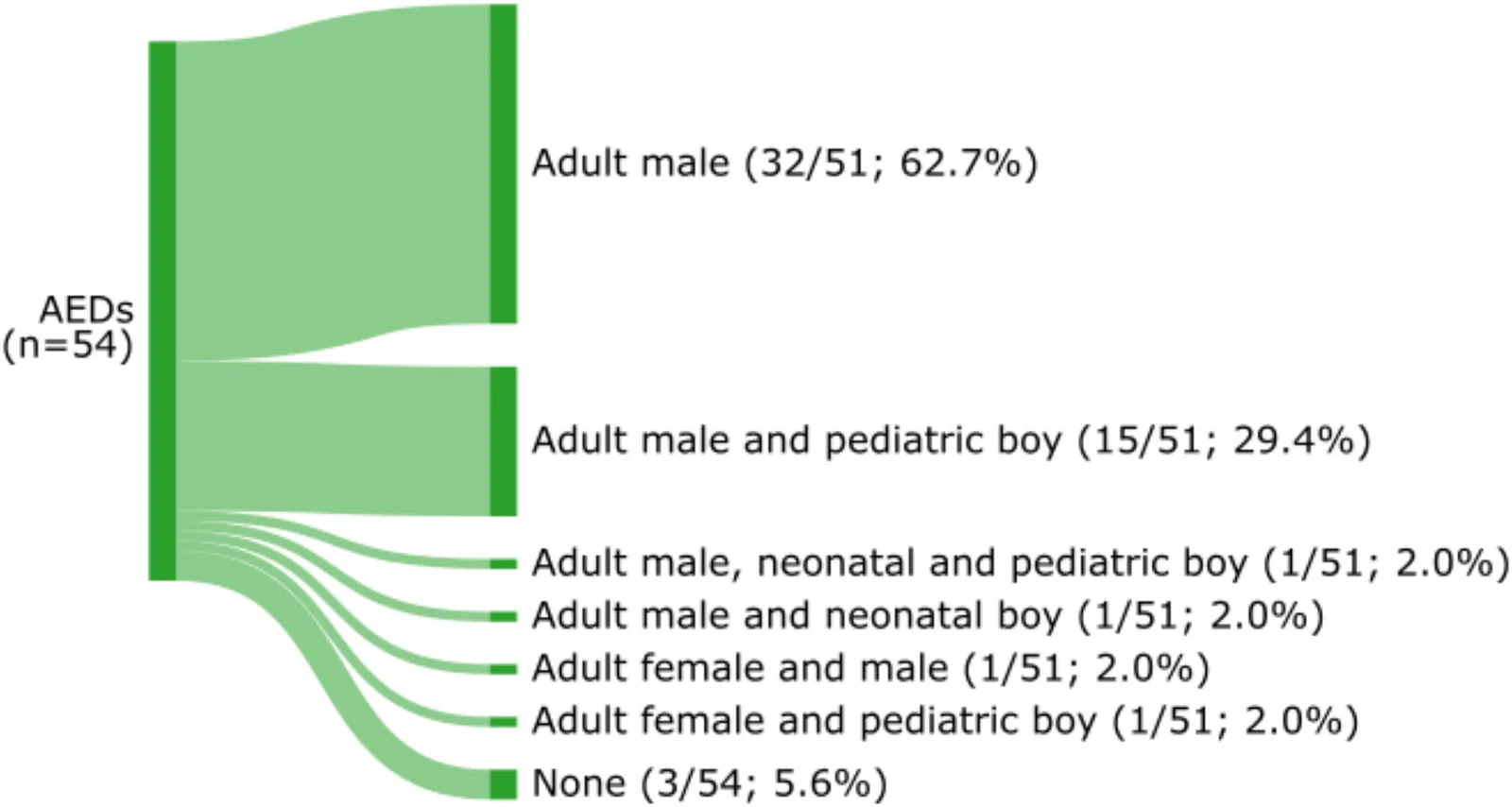
**Distribution of sex representation in pictograms displayed on AEDs**.

A total of 53 AED manufacturers worldwide producing 133 different AEDs were identified using the Google search engine. Of these, photographs of 54 AEDs from 25 AED manufacturers were obtained and reviewed (Fig. 1) (Supplementary 2). Three AEDs (3/54; 5.6%) did not feature any pictograms. Most AEDs displayed only adult male pictograms (32/51; 62.7%), while nearly one-quarter included both adult male and pediatric boy pictograms (15/51; 29.4%). Four configurations each appeared on a single AED: adult male, neonatal and pediatric boy (1/51; 2.0%), adult male and neonatal boy pictograms (1/51; 2.0%), adult female and male pictograms (1/51; 2.0%) (Fig. 2A), adult female and pediatric boy pictograms (1/51; 2.0%) (Fig. 3).

### Representation of sex in pad package pictograms

Out of 53 manufacturers and 41 distributors contacted, 23 responded, and 44 pad packages were included in the analysis (Fig. 1) (Supplementary 3). One pad package (1/44; 2.3%) did not feature any pictograms. Almost half of the pad packages displayed only adult male pictograms (21/43; 48.8%), while just over one-quarter included both adult male and pediatric boy pictograms (12/43; 27.9%). Smaller proportions featured adult male and pediatric girl pictograms (3/43; 7.0%) or adult male and neonatal boy pictograms (2/43; 4.7%). Adult female and male pictograms were present on two pad packages (2/43; 4.7%) (Fig. 2B). Rare configurations each appearing on a single pad package included adult female, adult male and pediatric boy pictograms (1/43; 2.3%); adult male, pediatric girl and neonatal boy pictograms (1/43; 2.3%), androgynous adult pictograms (1/43; 2.3%), and packages without any pictograms (1/43; 2.3%) (Fig. 3).

### Representation of sex in defibrillation pad set pictograms

**Fig. 4:**
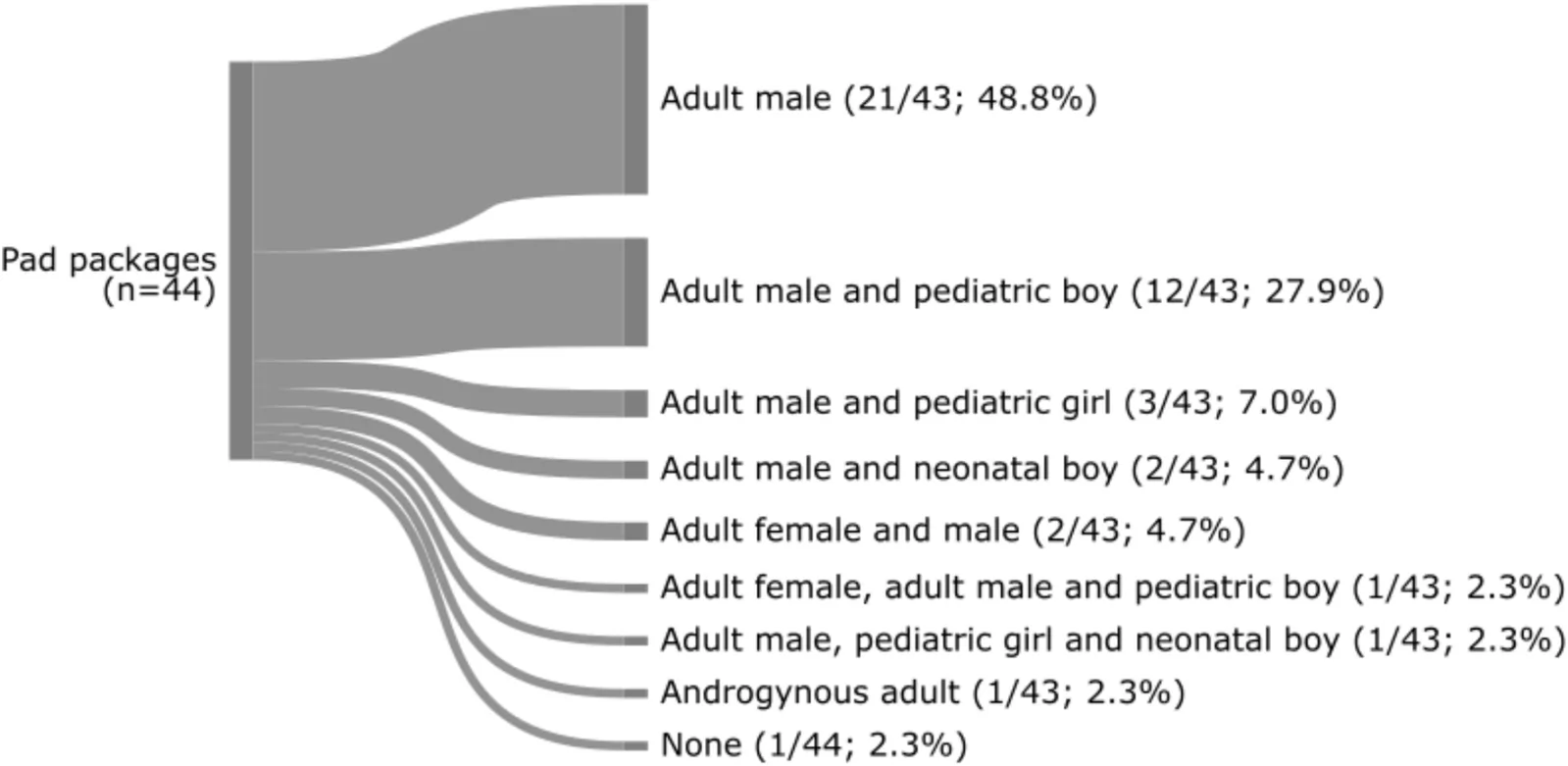
**Distribution of sex representation in pictograms displayed on pad packages**.

Among the 44 defibrillation pad sets examined (Fig. 1), more than half displayed only adult male pictograms (25/44; 56.8%), while just under one-quarter included both adult male and pediatric boy pictograms (10/44; 22.7%). Smaller proportions featured adult male and pediatric girl pictograms (4/44; 9.1%) or adult male and neonatal boy pictograms (2/44; 4.5%). Three configurations each appeared on a single set: adult female (on the apical pad only) and male pictograms (1/44; 2.3%) (Fig. 2C), adult female and pediatric boy pictograms (1/44; 2.3%) (Fig. 2C), and androgynous adult pictograms (1/44; 2.3%) (Fig. 4).

**Fig. 5:**
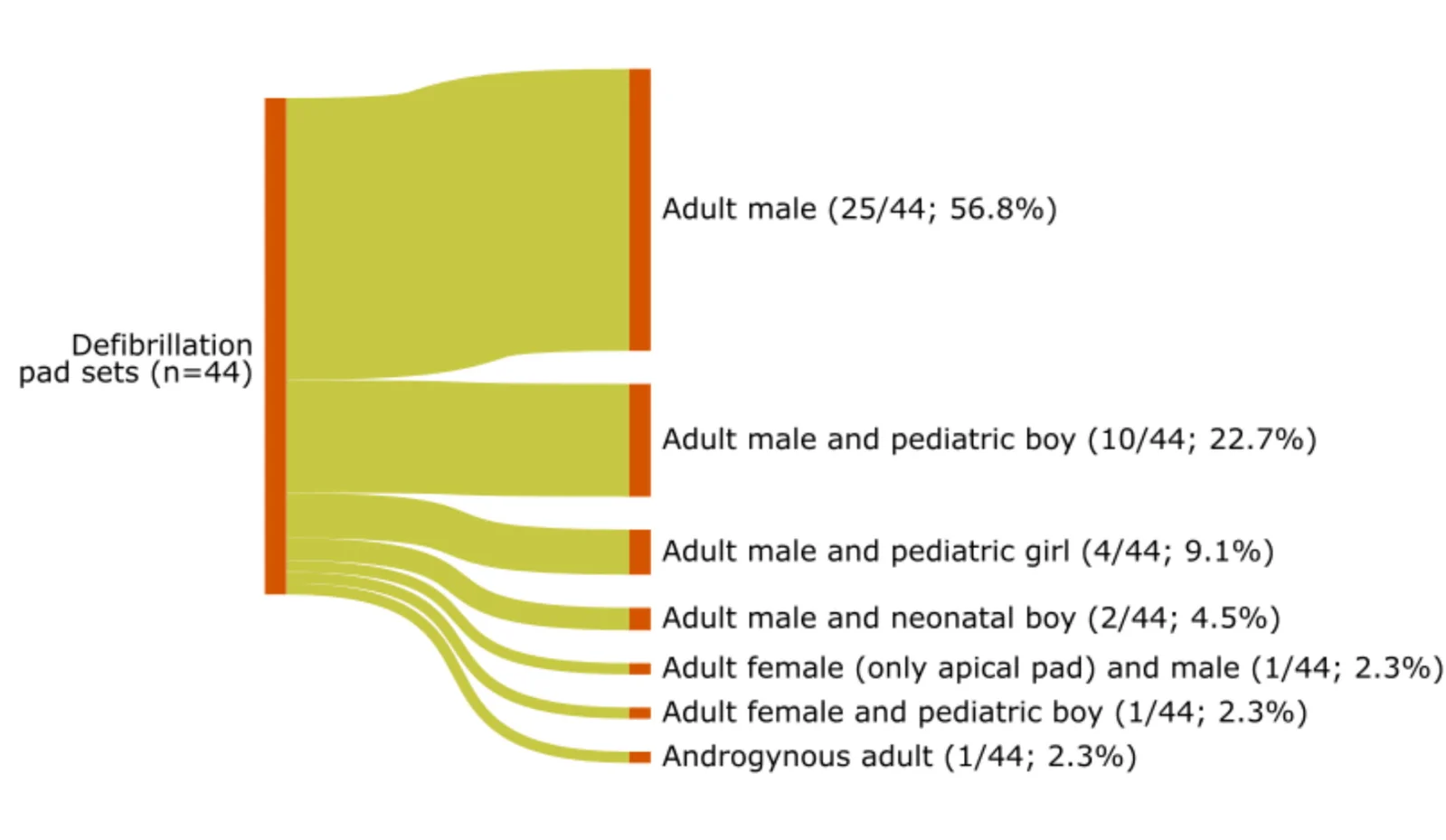
**Distribution of sex representation in pictograms displayed on defibrillation pad sets**.

Among all pictograms (*n* = 138) found on AEDs (*n* = 51), pad packages (*n* = 43), and defibrillation pad sets (*n* = 44), nearly all were computer-generated (*n* = 137; 99.3%), with only one pad package featuring a real photograph (*n* = 1; 0.7%) (Fig. 2 and Fig. 5). The agreement between investigators reviewing sex pictograms on the photographs was *almost perfect*, with a Cohen’s kappa value of 1.00.

## Discussion

This observational study of visual guidance displayed on AEDs, pad packages, and defibrillation pads revealed a striking predominance of adult male pictograms, with few female depictions overall. Androgynous pictograms were rare, and pediatric pictograms were overwhelmingly depicted as boys. These findings highlight a pervasive sex imbalance in the visual instructions represented for AED use across both adults and children.

The absence of adult female pictograms in AED instructional pictograms raises important concerns regarding both inclusivity and clinical accuracy. Because women also experience OHCA and require defibrillation, instructional materials that depict mostly male pictograms may be less representative and inclusive. However, prior research has shown that bystanders are less likely to initiate CPR on women.^6^, ^7^, ^8^, ^9^, ^10^, ^11^, ^12^, ^13^, ^14^, ^15^ The underlying reasons are not yet fully understood, but proposed explanations include fear of causing harm, concerns that chest exposure or contact could be misinterpreted as inappropriate or as sexual assault (even during a lifesaving procedure), and persistent sex-related stereotypes.^26^

The lack of female depictions on AEDs may reinforce the misconception that cardiac arrest occurs predominantly in men, leading to an under-recognition of women’s risk and the need for appropriate pad placement in female. In reality, wearing a bra^16^ or large breast tissue can affect optimal defibrillation pad positioning and therefore requires explicit visual guidance. This omission may represent a missed opportunity to normalize and encourage life-saving interventions for women. Notably, one of the newest ultralight AED models,^27^, ^28^ the AVIVE-AED (AVIVE Solutions, Inc., USA), departs from the long-standing convention of depicting only male pictograms. By including a female pictograms alongside a pediatric boy, it sends an important signal to well-established AED manufacturers worldwide about the feasibility and value of more inclusive imagery.

Similarly, the overwhelming representation of pediatric boys on the guidance, with few depictions of girls, may reflect an unconscious bias towards masculinity in medical device design. While cardiac arrest is less common in children, its incidence is reported to be similar in boys and girls.^1^, ^2^, ^4^ The absence of girls in the pictograms could inadvertently influence bystander perceptions of who “looks like” a cardiac arrest patient, potentially affecting recognition and response. Similar biases are observed in other areas of healthcare illustration, where male bodies are frequently depicted as the default model for medical imagery.^29^ Nearly all AED instructional pictograms we analyzed were computer-generated, with only a single photographic pictogram identified. While computer-generated imagery offers clarity, consistency, and removes distracting details, it may also reduce realism and make it harder for people to relate to. Photographic depictions that are diverse in terms of gender, age, body type, and ethnicity could improve recognition of real persons and help users visualize real-life applications. In our study, the sole photographic pictogram depicted white Caucasian individuals, which aligns with emerging literature highlighting a lack of racial diversity in medical materials.^30^, ^31^

Given these findings, the European Resuscitation Council (ERC) and national resuscitation councils could consider issuing a formal statement encouraging the use of AEDs and pad package/defibrillation pad illustrations that depict both female and male pictograms. While International Liaison Committee on Resuscitation has previously issued good practice statements to manufacturers regarding the inclusion of female training manikins, similar guidance has not yet been extended to AEDs or their packaging.^19^ highlighting a relevant gap in current recommendations. Such guidance and practice could help standardize inclusive visual representation across devices and training materials, aligning them with real-world demographics of OHCA patients. In addition, partnerships between female OHCA survivors, and public awareness campaigns as Bra Off Defib On,^32^ HeartCharged^33^, ^34^ or The CPR Bra^35^, ^36^ could play a relevant role in redesigning device imagery and instructions to better reflect female anatomy, address barriers to bystander intervention, and promote equitable care.^14^

Furthermore, regulatory bodies (e.g., U.S. Food and Drug Administration) and European Conformity frameworks already emphasize human-factors/usability and inclusive clinical evidence (e.g., sex-specific and standardized race/ethnicity data).^37^, ^38^ Building these frameworks, agencies and standards bodies (such as International Organization for Standardization) could further incorporate explicit inclusive-design requirements into device approval and certification, ensuring equity considerations are embedded in the appearance and usability of AEDs.^39^

### Limitations

Some limitations should be acknowledged. First, AEDs, pad packages, and defibrillation pads from manual or training devices were not included in this analysis. Since trainees should be instructed on AED use in both female and male patients, inclusivity in the pictograms of these devices is also important. Second, due to financial constraints, we were unable to purchase additional AEDs or defibrillation pads, which could have further enriched the dataset. In addition, participation may have been affected by the need for companies, manufacturers, distributors, and our professional networks and local sources to physically provide pad packages, and defibrillation pads to the investigators, together with the associated costs, which may have limited the response rate. Despite these constraints, our sample included products from several of the most widely used and researched^40^, ^41^ manufacturers, including ZOLL Medical Corporation (USA), Schiller Headquarters (Switzerland), Stryker Corporation, (USA), RadianQbio CO., Ltd (South Korea), Koninklijke Philips N.V. (Netherlands), and others (Supplementary 2 and 3). Third, we did not evaluate the correctness of pad placement depicted in the pictograms, as this was beyond the primary aim of the study. However, previous research^22^ has described instances of inaccurate pad positioning in instructional materials, and these studies are cited in support of this important issue. Finally, we did not include imagery of AEDs, pad packages, and defibrillation pads obtained solely from online sources, as done in a similar study,^23^ which could have increased the sample size. Instead, we collected and photographed the materials ourselves to ensure direct assessment. We are confident that including online pictograms would not have changed the results.

Our findings may serve as a foundation for a subsequent, hypothesis-driven study in which the intervention would involve guidance from resuscitation councils and collaboration with survivor- and advocacy-led initiatives to develop more inclusive AED and pad pictograms that represent both female and male patients and help reduce barriers to bystander intervention. In this respect, the topic is considered exploratory and forward-looking, rather than something that can be conclusively addressed within the current dataset.

## Conclusions

Our results suggest that AEDs, pad packages, and defibrillation pads predominantly depict male pictograms, with a near-total absence of female representations. Inclusive imagery is urgently needed to support recognition, proper pad placement, and equitable intervention during OHCA. Manufacturers and resuscitation councils could act to ensure AED design reflects all patients.

## Ethics declarations

None.

## Declaration of generative AI in scientific writing

We utilized DeepL and LanguageTool for spelling, grammar, and language refinement. After applying these tools, the authors reviewed and edited the content as necessary, assuming full responsibility for the final publication.

## CRediT authorship contribution statement

**Nino Fijačko:** Writing – review & editing, Writing – original draft, Visualization, Validation, Supervision, Resources, Project administration, Methodology, Investigation, Funding acquisition, Formal analysis, Data curation, Conceptualization. **Robert Greif:** Writing – review & editing, Writing – original draft, Visualization, Methodology, Conceptualization. **Guglielmo Imbriaco:** Writing – review & editing, Visualization, Methodology, Investigation, Conceptualization. **Priyanka Modi:** Writing – review & editing, Methodology, Investigation. **Roos Edgar:** Writing – review & editing, Visualization, Methodology, Investigation. **Kristin Alm-Kruse:** Writing – review & editing, Visualization, Methodology, Investigation, Funding acquisition. **Sergio Cazorla-Calderón:** Writing – review & editing, Writing – original draft, Visualization, Methodology, Investigation, Formal analysis, Data curation, Conceptualization.

## Funding

None.

## Declaration of competing interest

NF is a member of the European Resuscitation Council (ERC) Basic Life Support (BLS) Science and Education Committee. RG is ERC Director of Guidelines and International Liaison Committee on Resuscitation (ILCOR), and ILCOR Task Force chair for Education Implementation and Team; and member of the editorial board of Resuscitation Plus. GI is a member of the Scientific Committee of the Italian Resuscitation Council and the board of Aniarti, the Italian Association of Critical Care Nurses. RE is member of the young investigator editorial board of Resuscitation Plus. PA, KAK, and SCC, have no conflict of interest.
