# Supplementary material for: Representation of female and male pictorial instructions on automated external defibrillators, pad packages, and defibrillation pads
Source: Resusc Plus. 2026 Feb 11;30:101259. doi: 10.1016/j.resplu.2026.101259 (PMC13494629; doi:10.1016/j.resplu.2026.101259)
Supplement: Supplementary file 2 [file mmc2.docx]

**Supplementary 2:** List of AEDs.

1.	AVIVE-AED (AVIVE Solutions, Inc., USA)

2.	AED 3 (ZOLL Medical Corporation, USA)

3.	BIO-AED-I (BIOBASE, China)

4.	BeneHeart C2 (Shenzhen Mindray Bio-Medical Electronics Co., Ltd., China)

5.	BeneHeart L2 (Shenzhen Mindray Bio-Medical Electronics Co., Ltd., China)

6.	BeneHeart L1 (Shenzhen Mindray Bio-Medical Electronics Co., Ltd., China)

7.	BeneHeart C1 fully-automatic (Shenzhen Mindray Bio-Medical Electronics Co., Ltd., China)

8.	BeneHeart D1 (Pro) (Shenzhen Mindray Bio-Medical Electronics Co., Ltd., China)

9.	Corpuls AED (corpuls | GS Elektromedizinische Geräte G. Stemple GmbH, Germany)

10.	CellAED (Rapid Response Revival, Australia)

11.	Cardiagard-I (Allied Medical Ltd., India)

12.	Cardiagard-II (Allied Medical Ltd., India)

13.	CardioAid-1 (Innomed medical LTD, Hungary)

14.	Cardiolife AED-3100 (Nihon Kohden Corporation, Japan)

15.	CU-ER1 (CU Medical Germany GmbH, Germany)

16.	CardiAid AED (semi-automated) (Cardia International A/S, Denmark)

17.	CardiAid AED (automated) (Cardia International A/S, Denmark)

18.	FRED PA-1 (SCHILLER Headquarters, Switzerland)

19.	FRED PA-1 CardiAngel (SCHILLER Headquarters, Switzerland)

20.	FRED Easy (SCHILLER Headquarters, Switzerland)

21.	FRED easy life (SCHILLER Headquarters, Switzerland)

22.	FRED easyport plus (SCHILLER Headquarters, Switzerland)

23.	HeartSine Connected samaritan PAD 500P (HeartSine Technologies, Ltd., UK)

24.	HeartSine Connected samaritan PAD 350P, (HeartSine Technologies, Ltd., UK)

25.	HeartSine Connected samaritan PAD 360P, (HeartSine Technologies, Ltd., UK)

26.	HeartStart HS1 (Koninklijke Philips N.V., Netherlands)

27.	HeartStart FRx (Koninklijke Philips N.V., Netherlands)

28.	HeartOn A16-DS (MEDIANA Co., Ltd., Korea)

29.	iPAD CU-SP1 (CU Medical Germany GmbH, Germany)

30.	iPAD CU-SPR (CU Medical Germany GmbH, Germany)

31.	i3 (Ambulanc Shenzhen Tech. Co., Ltd., China)

32.	i5 (Ambulanc (Shenzhen) Tech. Co., Ltd., China)

33.	i7 (Ambulanc (Shenzhen) Tech. Co., Ltd., China)

34.	i9 (Ambulanc (Shenzhen) Tech. Co., Ltd., China)

35.	KAA-101 (Kaarya Medical Equipment Scientific Pvt LTD, India)

36.	LIFEPAK CRPlus (Physio Control, USA; now Stryker Corporation, USA)

37.	Lifepak CR2 (Physio Control, USA; now Stryker Corporation, USA)

38.	Lifeline AED (Defibtech LLC, USA)

39.	Lifeline AED PRO (Defibtech LLC, USA)

40.	Powerheart AED G3 (Cardiac Science Corporation, USA; now ZOLL Medical Corporation, USA)

41.	Powerheart AED G5 (Cardiac Science Corporation, USA; now ZOLL Medical Corporation, USA)

42.	PRO AED (LifePoint, Turkey)

43.	Radian HR‑501 (RADIANQbio Co., Ltd., South Korea)

44.	Reanibex 100 (Bexen cardio, Spain)

45.	Rescue Sam (Progetti s.r.l., Italy)

46.	Rescue Sam 4.0 (Progetti s.r.l., Italy)

47.	SMARTY SAVER (semi-automated) (AMI Italia, Italia)

48.	SMARTY SAVER automated (AMI Italia, Italia)

49.	SMARTY SAVER PLUS (semi-automated) (AMI Italia, Italia)

50.	SMARTY SAVER PLUS (automated) (AMI Italia, Italia)

51.	SMARTY SAVER GEO (automated) (AMI Italia, Italia)

52.	SAVER ONE (automated) (AMI Italia, Italia)

53.	SAVER ONE (semi-automated) (AMI Italia, Italia)

54.	ZOLL AED Pro (ZOLL Medical Corporation, USA)
