# Supplementary material for: Representation of female and male pictorial instructions on automated external defibrillators, pad packages, and defibrillation pads
Source: Resusc Plus. 2026 Feb 11;30:101259. doi: 10.1016/j.resplu.2026.101259 (PMC13494629; doi:10.1016/j.resplu.2026.101259)
Supplement: Supplementary file 3 [file mmc3.docx]

**Supplementary 3:** List of pad packages.

1.	Adult electrodes pads SAV-C0846 (AMI Italia, Italia)

2.	Adult electrodes pads SMT-C2001 (AMI Italia, Italia)

3.	Adult electrodes pads SMT-C1846 (AMI Italia, Italia)

4.	Adult electrodes pads SAV-C0599 (AMI Italia, Italia)

5.	Avive AED Pad Cartridge (Avive Solutions, Inc., USA)

6.	corPatch easy pro (Corpuls | GS Elektromedizinische Geräte G. Stemple GmbH, Germany)

7.	CPR Stat-padz (ZOLL Medical Corporation, USA)

8.	CPR Uni-padz (ZOLL Medical Corporation, USA)

9.	CPR-D padz (ZOLL Medical Corporation, USA)

10.	CUA1007S (CU Medical Germany GmbH, Germany)

11.	DDP-100 (Defibtech LLC, USA)

12.	DDP-2001 (Defibtech LLC, USA)

13.	Defibrillation Electrodes (Cardia International A/S, Denmark)

14.	Defibrillation Electrodes 9131 (Cardiac Science Corporation, USA; now ZOLL Medical Corporation, USA)

15.	Defibrillation Electrodes for CardiAid CT0207 (Cardia International A/S, Denmark)

16.	Defi-pad (AB Medica group S. A., Spain)

17.	Defi-pad 23DF2593 (AB Medica group S. A., Spain)

18.	Desfi-Dormo EDC-1020 (Telic group, Spain)

19.	Disposable defibrillation electrodes pads (Shenzhen Med-Link Electronics Tech Co., Ltd., China)

20.	Disposable multifuction electrodes for defibrillation R-S4-DFBAD01PRC (Progetti Medical)

21.	Disposable Pads P-740K (Nihon Kohden Corporation, Japan)

22.	DP Electrode Pads (Koninklijke Philips N.V., Netherlands)

23.	Electrodes for defibrillation MR60 (Shenzhen Mindray Bio-Medical Electronics Co., Ltd., China)

24.	Electrodes for defibrillation MR66 (Shenzhen Mindray Bio-Medical Electronics Co., Ltd., China)

25.	Eurodefipad adult lead out (Fiab, Italy)

26.	Eurodefipad adult radiotransparent (Fiab, Italy)

27.	HeartSine Pad-Pak (Heartsine Technologies Ltd., Northen Ireland)

28.	Heartstart smart pads II (Koninklijke Philips N.V., Netherlands)

29.	Heartstart smart pads III (Koninklijke Philips N.V., Netherlands)

30.	Life-az F946UW (Fiab SpA, Italy)

31.	NA (DefiSign, NA)

32.	P-303 AED pad (RadianQbio Co., Ltd., South Korea)

33.	Pad-Pak-03 (HeartSine Technologies, Ltd., UK)

34.	Pads EXLAED001B (Cardiac Science Corporation, USA; now ZOLL Medical Corporation, USA)

35.	Pads for defibrillation KSA0502: R100 pad (Bexen Cardio - Osatu s.coop., Spain)

36.	Pads for defibrillation TKL0311: Adult (Bexen Cardio - Osatu s.coop., Spain)

37.	PADS-A (MEDIANA Co., Ltd., Korea)

38.	PADS-APC (Hurev Co., Ltd., Korea)

39.	Philips HeartStart OnSite Adult Smart Pads – M5071A (HeartSine Technologies, Ltd., UK)

40.	Quik-pad (Physio Control, USA; now Stryker Corporation, USA)

41.	SavePads PreConnect (Primedic, Germany)

42.	Schiller FRED PA-1 Adult Electrode Pads 01-21-0040 (SCHILLER Headquarters, Switzerland)

43.	Skintach DF20 NC (Leonhard Lang GmbH, Austria)

44.	Stat-padz II (ZOLL Medical Corporation, USA)
